# Transactions of Albany County Medical Society, Semi-Monthly Meeting May 28

**Published:** 1873-08

**Authors:** F. C. Curtis

**Affiliations:** Secretary


ART. II.—*Albany County Medical Society. Semi-Monthly Meeting, held May 28th.* Reported by F. C. CURTIS, M. D., Sec'y.

The following resolution, adopted by the Common Council, was transmitted by the clerk of that body to the secretary of the county medical society:

*Resolved*, That the physicians of the city of Albany be requested, individually, to communicate to this board their opinion as to whether the water of the Hudson river is sufficiently pure and wholesome to be used to supply the city with water for the use of its inhabitants.

A meeting of the society was held to discuss the question proposed by the Board of Common Council. About twenty-five members were present, besides other gentlemen.

The President, Dr. VANDERVEER, opened the meeting by a statement of the subject for discussion, saying that it was one of grave importance to us all, and that we should discuss it in a calm, impartial manner. We were called upon to decide as to the fitness of the river water between here and Troy, not as to the cost or as compared with other sources.

Dr. DEVOL said that he had not very much practical knowledge of the river water. Water in general is the great solvent, taking up a great many of the matters found in nature and holding them in solution. He looked upon the river as the receptacle of a vast amount of refuse matter, which if retained in solution would certainly render it unfit for use. This seemed too plain to require any argument.

Dr. LEVI MOORE made the following remarks: Mr. President, the question before this society is one of great importance. We are asked as physicians if the water of the Hudson river is good in a sanitarian sense. The feasibility of this source of supply and its economic advantages have nothing to do with the question. The Hygenic aspect is the only one for us to consider. I trust that whatever action we shall take will be prompted by a sincere desire to secure to the public the greatest possible immunity from the germs of enteric and other diseases which we are so often able to trace to the use of impure, tainted or sewerage water. Whatever experts may say in commendation of sewerage water, even largely diluted with purer water and taken from a rapidly moving current, although it may be clear and sparkling, and possess no unpleasant taste or smell, yet the common instincts of our nature repel it as unfit for domestic use.

I need not bring to the attention of this society instances where typhoid fever, cholera, dysentry and other diseases have been clearly traced to the use of impure or sewerage water. These cases have been too numerous and too well authenticated to be doubted. The germs of disease are often too minute, too subtle to be weighed in the chemist's balance, or to be detected by his most careful analysis. Because he cannot detect any death-dealing poison in the limpid fluid, does he therefore presume to say that there is none, and that the water therefore is wholesome and good? Because the continued use of small does of an active poison does not kill at once, is it any the less a poison?

My own conviction is, that the water of Hudson River is entirely unfit for domestic use, receiving as it does the sewerage of our city, and within ten miles to the north of us, the sewerage of the cities of Troy and Cohoes and the village of West Troy. All this sewerage, together with that from the villages on the Hudson and Mohawk flows past our city and mingles with the sewerage which is here poured into the river. Admitting that the water of the Hudson River is passably good, and that its uses for domestic purposes are attended with but small risks in a hygenic point of view, still there are most cogent reasons why this source of supply should not be selected, I see in the no distant future a dense population, double, quadruple the present number in the manufacturing city of Troy and in the young city of Cohoes with its matchless water power. West Troy will have its population largely increased, and the beautiful hill sides between that point and this city are yet, I believe, to be thickly populated. All this drainage will flow into the Hudson River only a few miles above us, tainting its waters and rendering them each year more and more unfit for domestic use. The short-sighted policy of supplying other cities with water impregnated with sewerage has been so often recognized and deprecated that the very persistency with which we are shadowed with this practicable scheme for an abundant supply of pure water has something ominous about it. It is a notorious fact that even now the drainage from a large and rapidly increasing population flows into our present source of supply. This should be remedied at the earliest possible moment. The very fact that it has continued till it has become so serious a matter shows culpable carelessness somewhere. This society should take such action as the importance of the subject demands.

Dr. W. G. TUCKER read a paper on the analysis of river water. This, he said, contains organic matter generally in much greater proportion than spring water does, because of the surface drainage passing directly into rivers. Inorganic matters which they contain are of little moment except as they affect the hardness of the waters. Organic matters, from various sources, are diffused through the water, and being brought into contact with the oxygen contained therein, gradually change into carbonic acid, ammonia and nitrates. Thus the oxygen in water plays an important part in the purification of water. This is especially important where rivers receive, in addition to surface drainage, a large amount of sewage from towns, and in hot weather, especially, this natural process of purification is inadequate to prevent a putrefactive change.

The wholesomeness of water appears to be intimately connected with its state of aeration, as the proportion of oxygen is liable to be lessened by the decomposition of organic constituents.

Hudson River water was examined by Dr. TUCHER by what is known as the permanganate test, which is based on the oxidation of organic matters, this being preferred as the most delicate. During October and November last he made several examinations, with the result that about 4-10 of a grain was liberated to oxodize the organic matter in one gallon; city water examined at the same time required ⅛ of a grain per gallon. Early in February river water required 16-100 of a grain, city water 7-100; in April 33-100 of a grain was required for river water; city water was not tested at this time.

Prof. NICHOLS, of Troy, examined, by the same test, water from the river above Waterlord bridge and that in the Troy reservoir, and found a proportion in favor of the latter of 1 to 4. Other properties of the river water are a yellowish-brown or green color; it is turbid, with considerable sediment on standing; it contains ammonia in traces; its hardness is 3.9 deg., city water averaging 5 degs. Prof. MAYNARD, of Troy, found 4.94 grains of solid matter per gallon, 1.08 grain being organic, and he expresses the opinion that the water of rivers receiving sewerage are unfit for domestic use.

The examination of water made by Prof. CHANDLER, Dr. TUCKER said, does not fairly represent the character of the water, because the sample was taken from under the ice in the latter part of the winter, when, being at a low temperature, it would hold less in solution than during the summer. There is also at that season much less opportunity for foreign matters to get into the water. Prof. Chandler's report gave, however, at this season, 7-10 grain per gallon of organic matter.

Dr. TUCKER believed that taking river water for a city supply involved a great risk. A single analysis is not sufficient to determine the purity of the water, but it must be examined at different seasons, states of the weather, and stages of the river. The examinations already made do not warrant its use.

Dr. FREEMAN said that he did not understand that the question was limit‹ d to the fitness of the water taken from a point opposite the city. He thought the river ought to be used as our source of supply, being reliable, permanent and abundant at all seasons. It is supplied at its source and along its course by mountain springs. It would certainly be pure enough if taken from a point north of Cohoes; and, besides, from this elevation it would supply the city without pumping.

Mr. G. MICHAELIS, by a vote of invitation, read a paper as follows:

GENTLEMEN: It is not the place here to give you a review of the chemical and mechanical properties of the water; it is another point altogether which we have to take in consideration, and that is the purity or impurity of it.

All waters, river water as well as well water are powerful solvents of the ingredients of the soil, and contain therefore a variable quantity of the salts which are frequently found in it. They are principally chloride of sodium, sulphate of lime, magnesia salts, and so forth, which give the water, especially when it contains carbonic acid, a refreshing taste, and there is no doubt that they have a healthy influence on the human body.

But all waters, no matter whether river or well water, contain more or less organic matter, and it is supposed to a certain point certified that that the latter is obnoxious to the human body and produces sickness. The sources wherefrom the organic matter in rivers come are well known; they are principally the sewage and manure of the towns and villages through which it flows, consisting of almost everything, urine, fæces, dead animals and the decay of the plants of the stream; a water thus impregnated is undoubtedly not very healthy. On the other hand, speaking of well water, we must state that wells in cities are very quickly spoiled by infiltration of manure, and thus become bad.

I made an experiment which showed this in the spring of 1866, when cholera was imported from Russia to Germany. This raised general alarm throughout the country, and more so since some prominent physicians supposed that the ground water had something to do with the spreading of the disease. I was at that time in Posen, a city of about 65,000 inhabitants, in the chemical laboratory of Dr. Mankiwich occupied as his assistant. This gentleman received an order from the city authorities to analyze the water of the city wells. A thorough investigation proved that nearly one third were in a bad condition and entirely unfit for use, but some of the wells, even those with a large amount of organic matter appeared clear to the eye, while others had a good taste and very little smell. An analysis made of the water of the river Warthe proved that it was in at least as good a state as the wells.

We must understand that there are two distinctly different organic matters in the water, such are those dissolved in the water and those only mechanically suspended in it. The mechanically suspended matter can be easily eliminated by a careful filtration, as filtering through gravel or sand and charcoal, but the greater part of organic matter is dissolved in the water and cannot be separated by mere filtration. It is beyond doubt that the dissolved organic matter is the source of the bad odor and the yellow, brownish color. Pure water is colorless. Now the question is, whether there is a possibility of eliminating the dissolved organic matter or not? This question was satisfactorily answered by Prof. Medlock. This gentleman was requested in the year 1856 by the Amsterdam water company in Holland to analyze their water and to find the cause of the bad smell and fishy taste (I wish you to understand that the water in Amsterdam is let through iron pipes). Medlock received five samples of water for analysis, four of them from pipes in the city and one before the water entered the reservoir of the company.

In the water from the works before it came in contact with the iron reservoir and pipes this chemist found 0.95 grains of iron while in the other samples the iron was reduced to an unweighable trace; thus instead of taking up iron from the service pipes it was not alone diminished but even all precipitated. Notwithstanding the almost entire precipitation of the iron actually in solution in the water which had passed the iron pipes it formed an objectionable red deposit on standing, while the water from the works before entering the pipes holding in solution nearly ½ grain of iron per gallon formed no deposit. He examined the deposit chemically and microscopically with the following result. On motion the precipitate charred and was almost entirely consumed, showing them that it consisted of organic matter; under the microscope the deposit was seen to consist of plants in various stages of decay, many of the fibres had even retained a perfectly organized structure. He remembered the important fact established by Shonbein that copper and platinum can convert ammonia into nitrous acid, and he found that iron also transforms ammonia into nitrous acid and by further investigation he found that strips of sheet iron placed in water containing ammonia or organic matter capable of yielding it act almost as quickly and well as the metal in a finely divided state.

Now he made different analyses with water brought in contact with iron, and water in no contact with iron, and the result was, that while the water which was not brought in contact with iron contained 2.10 organic matter and 0.95 iron, the other contained only an unweighable trace of both, which showed plainly that the organic matter contained in the water was either decomposed or thrown down (rendered insoluble by contact with iron), and actually this water when filtered, was clear, of good taste, had no smell, and was free from organic matter.

All organic matter either contains nitrogen or is non-nitrogenous. The latter consists almost always of carbon, oxygen and hydrogen, and is very quickly, when no more under the vital influence, converted into carbonic acid and water. The nitrogenous matter is mostly of animal origin, and consists of nitrogen, oxygen, hydrogen, and very often sulphur and phosphorus, and is dissolved or suspended in water, very rapidly decomposed into gases very offensive to taste and smell, and undoubtedly very obnoxious. But all these are immediately destroyed by the presence of nitrous acid. Medlock proved, as before stated, by a series of subsequent analyses that iron produces nitrous acid by its action on the nitrogenous organic matter, which is the most destructive power nature has. Muspratt calls it "Nature's scavenger." This chemist found as a general result that by allowing water to be in contact with a large surface of iron, in about 48 hours every trace of organic matter was either destroyed or rendered insoluble, in which state it could be purified effectually by filtration.

I did the same with our Hudson river water and obtained the same results. After subjecting the most fifthy water to the influence of iron sheets for 48 hours, I found that it was almost entirely freed of all organic matter.

I submit to the honorable society some samples of Hudson water which I treated in a similar manner; and it can be easily seen that the iron sheets have had some influence on it; there is a decided deposit.

[Specimens of water treated with iron sheets, which appeared to be perfectly pure, were exhibited.]

It would be perhaps of some value if similar researches should be made, and in case the results should be favorable, it would not make any difference wherefrom we receive the water as long as we are able to purify it in such a manner as to render it a wholesome beverage for the community.

The experiment shows the existence of organic matter in the river water.

Dr. Sabin, of West Troy, said that analyses which had been presented show the water opposite the city not sufficiently pure for use; he thought the best plan was to take water from the river just below the State dam above Cohoes and Green Island. At this point it is purified by running over the dam.

Dr. Wm. Hailes spoke of experiments which had been made to purify water, showing it to be a fallacy that water is purified by flowing over a dam. Pure water was mixed with fæcal matter in vessels, and poured day after day over a rough board, allowing it thereby to be most intimately mixed with air, and at the end of two weeks it was still found impure. It is also a fallacy that digging wells and so filtering purifies water, as it has been proved that filtering does not purify it. The germs of disease are not removed by it. He thought that river water here, and even at the State dam, is unfit for use.

Dr. Crothers followed at some length. He assumed hypothetically that the river water was better in quality than that we are now using. The impurities the river water contains are disseminated over a wider surface, comparatively, than those in the water we are using. Nevertheless, he maintained that, as water is a medium for spreading disease, it was unfit for ordinary use. He said the bad condition of the water we now use is caused by a neglect to keep out impure drainage and allowing it to remain in shallow ponds never cleaned. He held that the impurity of our water affected the health of the city and was a constant threat of epidemic diseases. He maintained these points at much length.

Dr. James S. Bailey remarked that he had been much interested in what had been said, especially in the careful analyses of the chemists; but it had occured to him that they might be more technical than practical. The air we breathe might by analysis prove to contain deleterious elements, but still we live and apparently enjoy health; so with river water; it undoubtedly contained organic matter, but when taken into the stomach does not prove deleterious to animal economy. He had known persons, in fact whole families to drink river water constantly with impunity, and from southern waters which were known to be more deeply impregnated with earth and vegetable matter than the water of the Hudson. He had known families to use water from stagnant pools covered with green scum, by brushing this aside, and the water, when used, did not produce unhappy results. We well know without the aid of chemistry that the waters of the Hudson about this city impregnated with sewage is unfit for culinary or drinking purposes; but it seems that it can be obtained above the city. We need the quantity as well as the quality, and he could not see where it could be obtained unless from the Hudson. There are processes in nature—chemical processes, really—by which water is purified, which is exemplified in the commingling of the waters of the Mississippi with those of the Ohio at Cairo, one coming from the limestone country, and the other ladened with earthy matter, which when united had the desired effect of producing a chemical change, in fact a fil tering process, and it is a fact well known by river men that water in this vicinity is unusually pure and wholesome.

Dr. Craig said that he believed the Hudson river water was purer at its source than any other. It had been used for very many years for drinking purposes; ships going to sea came up into the Hudson river and took their supply. He said the Hudson river contains a volume of water four times as large as the river Thames, and the city of London is supplied from the latter. He thought the society should not be hasty in its judgment or too ready to condemn this source of supply.

Dr. Davis remarked—The statement made here this evening that river water has been used with impunity and is therefore innoxious and wholesome reminds me of accounts given by travelers of some barbarous tribes who habitually eat their food in the most offensive state of putrefaction and decay. Civilization with its overtasked brain and nervous system can ne ver withstand such vile and unwholesome food. No more can we long endure the baneful influence of river water filled with putrescent vegetable and animal substances drained from an extensive region of country, and to which are added the sewerage of cities, holding in solution vital and putrefactive animal poisons without subjecting ourselves to the natural and inevitable consequences of taking into our systems those active morbific agents which have ever proved most destructive to humanity. The best water is that derived from springs, which have their original reservoirs deep in the earth, or from those lakes having a natural outlet but holding temporarily the water from these springs. It is true the contents of these reservoirs has once been surface water but by percolating through the soil, sand and gravel beds has thus been filtered and freed from every noxious element. Next to these waters in purity are those upland streams that flow from them. As we leave these sources water becomes less pure till we reach those large rivers that flow through the valleys and receive numerous tributaries with extensive surface drainage. These rivers are the common receptacle of every unclean thing, and when the shores are covered with the dense population of large towns and cities, the sewerage of every dwelling, prison, hospital and sick room is mingled in the general mass of its impure waters, containing epidemic, contagious and infectious elements. Chemistry has its range and its limits, and has never been able to detect those atenuated and subtle principles that produce the desolating ravages of cholera, cerebro-spinal meningitis, small pox, fevers and numerous other diseases, and yet of their existence no truth in the whole history of medicine is better established. This subject, with great property, addresses itself to the medical profession, and I hope we shall show ourselves no less worthy to serve the interests of the public in the preservation of health than for its restoration when lost. The question with which the water commissioners have honored us is not the merits of river water as compared with our present supply, but "is the water of the Hudson river pure and wholesome for domestic use."

I hope this society will place upon it their disapprobation, and will express with emphasis their answer, no.

Dr. Wm. H. Bailey said that he did not believe any source equal to the Hudson, when we take all things into consideration. It is not necessary to take from a point opposite the Hospital or even between here and Troy, although he believed that pure water might be found below the latter place. But we may at least take water above these sources of sewerage. Water does not retain organic matter for any length of time, but it purifies itself. The upper Hudson flows through an elevated country and its sources are healthy. We hoped the discussion would not be concluded without a second meeting being held, in order to get a fair view of the opinion of the society.

On motion, adjourned.

